# A comparative study of growth: different body weight trajectories in three species of the genus *Eublepharis* and their hybrids

**DOI:** 10.1038/s41598-018-19864-3

**Published:** 2018-02-08

**Authors:** Daniel Frynta, Jitka Jančúchová-Lásková, Petra Frýdlová, Eva Landová

**Affiliations:** 1 Department of Zoology, Faculty of Science, Charles University, Viničná 7, CZ-12844 Prague 2, Czech Republic; 2 National Institute of Mental Health, Topolová 748, CZ-25067 Klecany, Czech Republic

## Abstract

An extensive research effort is devoted to the evolution of life-histories and processes underlying the variation in adult body weight; however, in this regard, some animal taxa remain neglected. Here we report rates and timing of growth recorded in two wild-derived populations of a model lizard species, *Eublepharis macularius* (M, W), other two related species, i.e., *E. angramainyu* (A) and *E. sp*. (D), and their between-species hybrids. We detected clear differences among the examined species/populations, which can be interpreted in the terms of “fast – slow” continuum of life-history strategies. The mean asymptotic body size was the highest in A and further decreased in the following order: M, W, and D. In contrast, the growth rate showed an opposite pattern. Counter-intuitively, the largest species exhibited the slowest growth rates. The final body size was determined mainly by the inflexion point. This parameter reflecting the duration of exponential growth increased with mean asymptotic body size and easily overcompensated the effect of decreasing growth rates in larger species. Compared to the parental species, the F_1_ and backcross hybrids exhibited intermediate values of growth parameters. Thus, except for the case of the F_2_ hybrid of MxA, we failed to detect deleterious effects of hybridization in these animals with temperature sex determination.

## Introduction

Body size is a crucial parameter determining the ecological and evolutionary attributes of animals^1,2^. Its phenotypic variation may be caused by both genetic and environmental components^3^. As a result, body size contributes to the fitness and varies substantially among individuals, populations, and species^4–6^. The final body size is not just a static trait, but is a product of an ontogenetic trajectory typically involving the growth process.

Growth trajectories are perfectly understood in fast-growing avian species^7^. A collection of datasets covering the entire period of growth is sometimes extremely laborious and time-consuming, especially in case of species with slow ontogenetic trajectories and/or indeterminate growers (but see in squamates reptiles^8–14^ and in fishes^15–19^). This subsequently simplifies the description of body growth as a function of growth increments (typically used in agri- and aqua-culture).

The growth trajectories typically consist of two major components contributing to the final body size. In a typical case, these are an intrinsic growth rate and a duration of exponential growth period. The latter one is not estimated as a separate parameter by some widely-used theoretical growth models (e.g. von Bertalanffy, West production model), which are applicable even when the data points do not cover the whole course of ontogeny^20,21^. The logistic growth model fits the empirical data concerning a detailed description of growth trajectories and produces the required information about the duration of exponential growth period further referred to as an inflexion point. The estimates from this model are the growth rate, inflexion point, and asymptotic body size^22^. Both the growth rate and the growth duration may contribute to the final body size and are inherently inter-correlated.

Besides the growth trajectories and their components (parameters), life-history variables comprise of the body size, maturation, longevity, mortality rates, reproductive investments, etc. In many animal taxa, these variables are tightly inter-correlated and arranged along a common gradient, typically forming an axis from slow to fast life-histories^23,24^. Thus, the composite measure of life-histories from a multivariate data set is used to classify the position across the current concept of the “fast-slow” continuum (e.g.,^24–27^). Even in the absence of complex data, it is possible to estimate an approximate position of individual species/populations on this axis according to a limited number of reliable life-history variables. In this respect, growth rates and/or duration of exponential growth may be, under some circumstances, helpful.

In our study, we monitored three closely related species of eyelid geckos of the genus *Eublepharis*. During the last century, leopard gecko (*Eublepharis macularius*, Blyth, 1854) became a laboratory animal as well as a captive bred pet. It is routinely used as a model species for studies of incubation temperature and its hormonal consequences influencing brain development^28–31^, antipredatory strategies, etc.^32,33^. Eublepharid geckos (Eublepharidae) vary considerably in body size – the largest species *E. angramainyu* (Anderson and Leviton, 1966) is more than 20 times heavier than the smallest *Coleonyx brevis* (Stejneger, 1893). Thus, the family was repeatedly used as a model for studies dealing with the evolution of body size^34^, parental investment^35,36^, growth^37^, allometries of cell size, DNA content, and metabolism^38–40^.

Growth rates and trajectories may properly reflect the animal’s life-history strategy only if these parameters were solely determined by the underlying trade-offs and the corresponding strategic decisions concerning an investment. Growth may be constrained by fitness (performance, health status) of the animal. In this respect, the efficiency of growth can be useful during the monitoring of the processes that are suspected to cause deleterious effects.

We compared the growth parameters to explore the effect of experimental crossing of the species/populations of the eublepharid geckos. The view on the real size of the effect of hybridization on fitness is still controversial. Both negative and positive outcomes were associated with hybridization in natural and experimental conditions^41–43^; for reviews, see^44–47^. Historically, natural hybridization was considered as exceptional and erroneous events^48^, but the current increase of literature concerning the importance of hybridization for both speciation and adaptation implies the opposite^44,47,49–51^. Genomic and epigenetic insights into the molecular bases of heterosis are indicating that the role of natural hybridization is important in the formation of new species. Because of exceptional time demands, experimental studies dealing with hybridization covering the observation of real parameters of fitness (fertility, viability, body growth) are still very scarce^43,52–54^.

The aims of our study were 1) to compare the growth parameters of leopard geckos to demonstrate contrasting life-history strategies of selected parental species/populations; and 2) to compare the growth parameters of parental species with parameters of F_1_ and F_2_ hybrids and subsequent backcrosses to reveal the putative beneficial (heterosis in F_1_ generation of hybrids) and/or deleterious (incompatibilities leading to segregation load in F_2_ and backcrosses) effects of hybridization on fitness.

## Results

**Figure 1:**
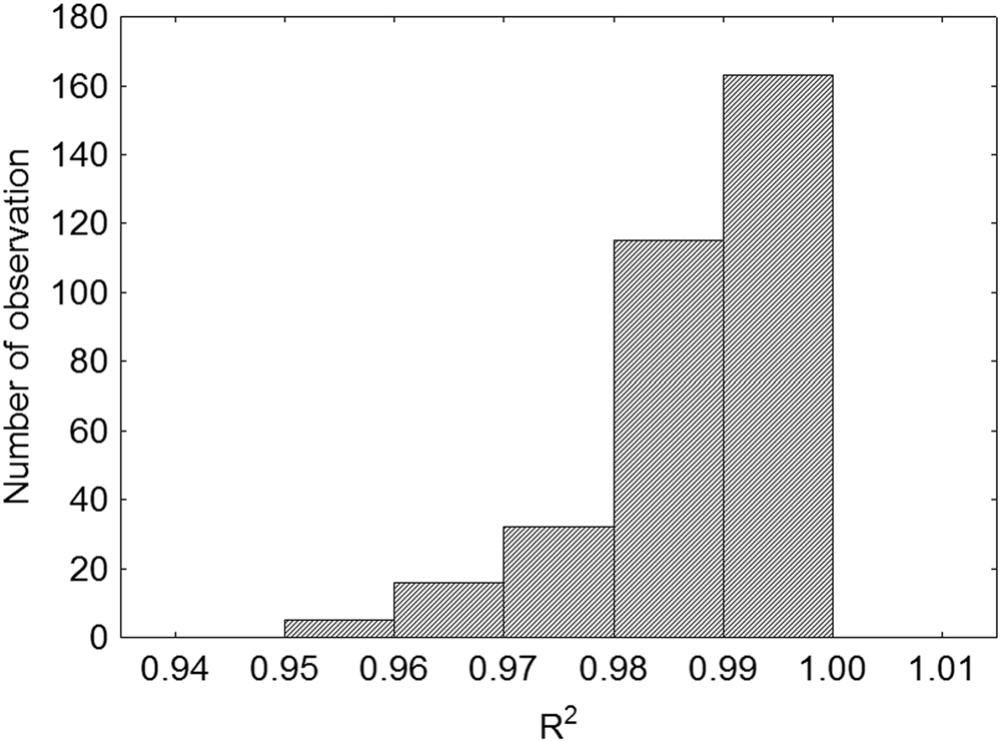
Histogram of variance explained (R^2^) by a logistic regression model for all studied individuals.

The estimated growth parameters for distinct populations/species, their F_1_ and F_2_ hybrids, and backcrosses are presented in Table 1. The logistic regression model fits well our longitudinal growth data (Table 1 and Fig. 1).

**Table 1:** The estimated values (mean ± SE) of the asymptotic body mass a (g), growth rate K and inflexion point T (days), and variance explained by the model (R^2^) with a number of individuals (N) for the examined species, hybrids, and backcrosses of eublepharid geckos.

Generation	Species/population	a ± SE	K ± SE	T ± SE	R^2^ ± SE	N
P	A	101.135 ± 4.782	0.0055 ± 0.0021	381.17 ± 22.21	0.989 ± 0.003	6
P	W	39.461 ± 1.852	0.0153 ± 0.0008	167.68 ± 8.6	0.985 ± 0.001	40
P	D	30.44 ± 1.876	0.0194 ± 0.0008	140.79 ± 8.71	0.991 ± 0.001	39
P	M	49.188 ± 1.64	0.0144 ± 0.0007	182.96 ± 7.62	0.987 ± 0.001	51
F_1_	WxD	35.851 ± 4.782	0.0149 ± 0.0021	145.14 ± 22.21	0.98 ± 0.003	6
F_1_	MxW	43.764 ± 2.928	0.0151 ± 0.0013	160.95 ± 13.6	0.988 ± 0.002	16
F_1_	MxA	78.501 ± 2.254	0.0108 ± 0.001	264.14 ± 10.47	0.981 ± 0.002	27
F_1_	MxD	40.484 ± 2.687	0.0137 ± 0.0012	168.37 ± 12.48	0.986 ± 0.002	19
F_2_	WDxWD	34.243 ± 4.427	0.019 ± 0.002	136.59 ± 20.57	0.991 ± 0.003	7
F_2_	MWxMW	38.483 ± 2.841	0.0187 ± 0.0013	137.92 ± 13.2	0.989 ± 0.002	17
F_2_	MAxMA	36.546 ± 11.713	0.0102 ± 0.0052	178.52 ± 54.41	0.984 ± 0.008	1
F_2_	MDxMD	36.87 ± 3.532	0.0195 ± 0.0016	135.19 ± 16.41	0.992 ± 0.002	11
B	MAxM	45.199 ± 3.704	0.0131 ± 0.0016	155.54 ± 17.21	0.98 ± 0.003	10
B	MxMA	57.347 ± 3.024	0.0115 ± 0.0013	202.94 ± 14.05	0.984 ± 0.002	15
B	MxMD	40.595 ± 8.282	0.0206 ± 0.0037	128.34 ± 38.47	0.991 ± 0.006	2

Abbreviations: (P) parental generation, (F_1_) the first and the second (F_2_) filial generation hybrids, (B) the first-generation backcross, (M) the parental generation of the yellow population of *E. macularius*, (W) the parental generation of the white population of *E. macularius*, (A) the parental generation of *E. angramainyu*, (D) the parental generation of the dark population of the genus *Eublepharis*, (MxA) the first-generation hybrid, a mother of the yellow population of *E. macularius* and a father of the *E. angramainyu*, (MxD) the reciprocal first-generation hybrid, a mother/father of the yellow population of *E. macularius* and a mother/father of the dark population of *E. sp*., (WxD) – the first-generation hybrid, a mother of the white population of *E. macularius* and a father of the dark population of *E. sp*., (MxW) – the reciprocal first-generation hybrid, a mother/father of the yellow population of *E. macularius* and a mother/father of the white population of *E. macularius*, (MAxMA) - the second-generation hybrid, both parents are F_1_ hybrids of the yellow population of *E. macularius* and *E. angramainyu*, (MDxMD) the second-generation hybrid, both parents are F_1_ hybrids of the yellow population of *E. macularius* and the dark population of *E. sp*., (WDxWD) the second-generation hybrid, both parents are F_1_ hybrids of the white population of *E. macularius* and the dark population of *E. sp*., (MAxM) the first-generation backcross with the yellow population of *E. macularius*, a mother is an F_1_ hybrid of the yellow population of *E. macularius* and *E. angramainyu* and a father belongs to the yellow population of *E. macularius*, (MxMA) the first-generation backcross with the yellow population of *E. macularius* (reciprocal to previous), a mother belongs to the yellow population of *E. macularius* and a father is an F_1_ hybrid of the yellow population of *E. macularius* and *E. angramainyu*. First, there is always an abbreviation for a female, followed by the one for a male with a cross (x) between.

### Comparisons among parental species/populations

**Figure 2:**
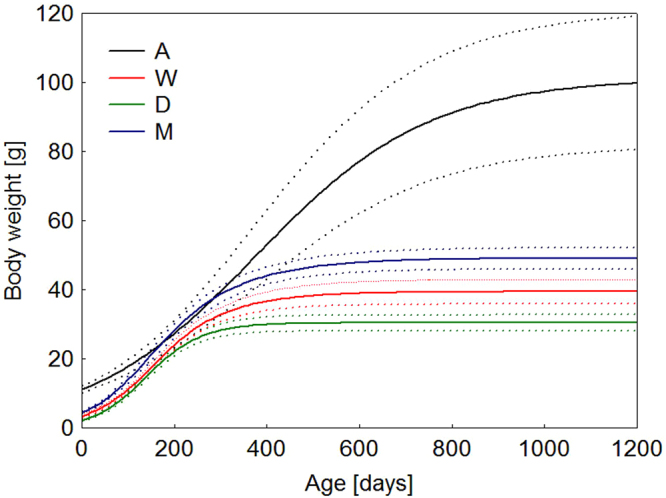
Mean body weight as a function of age predicted by the logistic growth model in studied species of eyelid geckos. Growth parameters were estimated from pooled records of either species/populations. Dotted curves are ± 95 confidence intervals for means of studied species/populations. Abbreviations: (M) yellow population of *E. macularius*, (W) white population of *E. macularius*, (A) *E. angramainyu*, (D) dark population of *E. sp*.

Growth parameter *a* significantly differed (Table 1) among distinct species/populations (ANOVA: F_3,132_ = 88.337, P < 0.001). Moreover, *E. angramainyu* exhibited significantly lower growth rate *K* (ANOVA: F_3,132_ = 16.3791, P < 0.001) and bigger inflexion point *T* (ANOVA: F_3,132_ = 37.057, P < 0.001) than all of the other species/populations. The growth parameters revealed from the logistic regression model were inter-correlated. The asymptotic body weight (*a*) was closely correlated to parameter *T* (r = 0.64, 0.75 and 0.65 for yellow, white, and dark species/populations, respectively). No such correlation was found between *a* and *K* parameters. The whole course of body growth of distinct species/populations is illustrated by Fig. 2.

**Figure 3:**
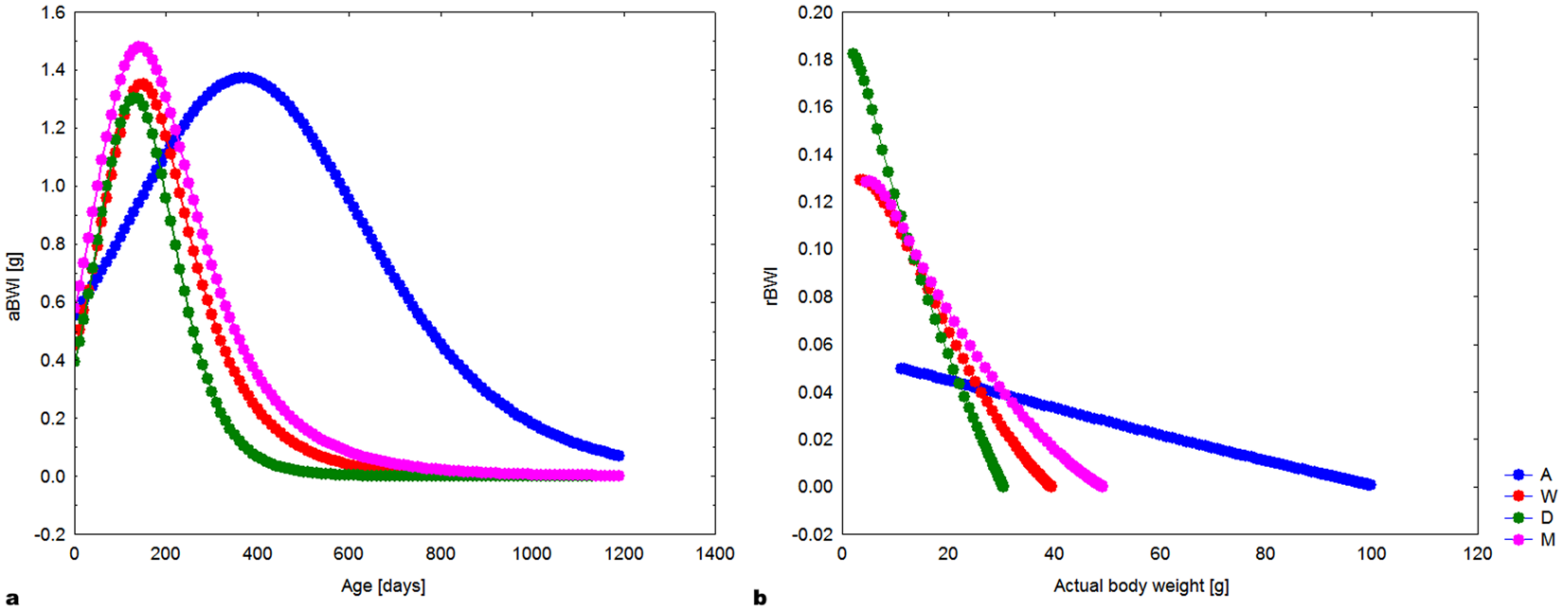
Absolute body weight increments (aBWI) as a function of age (A) and relative body weight increments (rBWI) as a function of actual body weight for distinct species/populations (B). Abbreviations: (M) yellow population of *E. macularius*, (W) white population of *E. macularius*, (A) *E. angramainyu*, (D) dark population of *E. sp*.

Changes in mean absolute and relative body weight increments (computed from real body weighting) during the post-hatching ontogeny are shown in Fig. 3.

### Comparison among parental species (*E. angramainyu* and *E. macularius*), their F_1_ and F_2_ hybrids, and backcrosses

**Figure 4:**
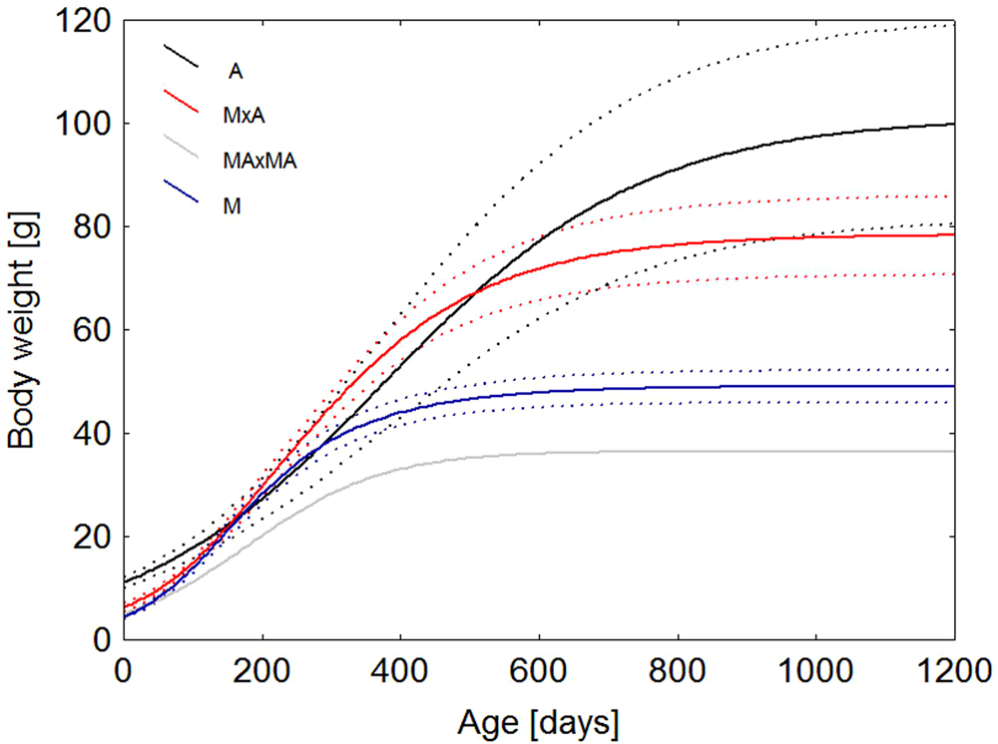
Mean body weight as a function of age predicted by the logistic growth model in parental species of yellow population of *E. macularius* (M) and *E. angramainyu* (**A**) and their F_1_ (MxA) and F_2_ (MAxMA) hybrids. Growth parameters were estimated from the pooled records of either species and the hybrids. Dotted curves are ± 95 confidence intervals for means of studied groups. Note the growth curve of F_1_ hybrids (N = 27), which is between the parental species’curves and the poor growth of F_2_ hybrid.

Growth parameters significantly differed among parental species of A and M and their F_1_ hybrids (ANOVA: *a*: F_(2,81)_ = 57.725, P < 0.0001; *K*: F_(2,81)_ = 10.0467, P = 0.000127; *T*: F_(2,81)_ = 24.9932, P < 0.0001). F_2_ hybridization was not successful (except for one individuum, which had poor body growth with the lowest prediction of parameter *a* = 36.546 g) in comparison with the parental population and F_1_ hybrids. The course of body growth of the parental species and F_1_ and F_2_ hybrids is depicted in Fig. 4. The asymptotic body weight significantly differed in both backcrosses (MAxM and MxMA) from the parental species of *E. angramainyu* and F_1_ hybrids (ANOVA: F_(4,104)_ = 29,771, P < 0,0001). The estimations of asymptotic body mass were similar for both backcrosses (see Table 1).

### Comparison among parental species (yellow and white *E. macularius* and dark *E. sp*.) and their F_1_ and F_2_ hybrids

**Figure 5:**
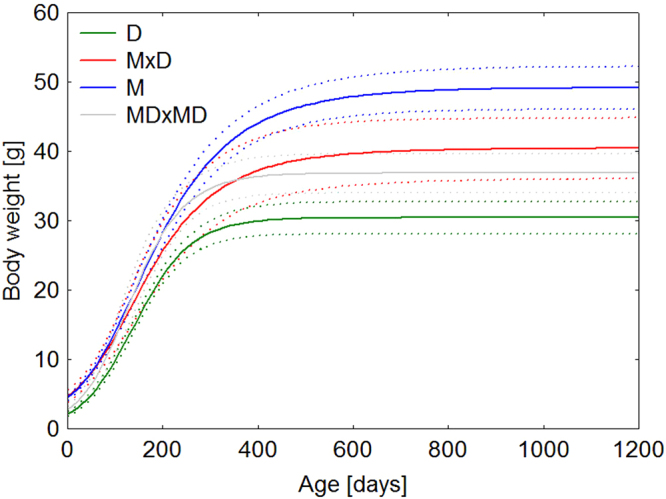
Mean body weight as a function of age predicted by the logistic growth model for parental species of the yellow population of *E. macularius* (M), the dark population of *E. sp*. (D), F_1_ (MxD) and F_2_ (MDxMD) hybrids. Growth parameters were estimated from pooled records of either species and the hybrids. Dotted curves are ± 95 confidence intervals for means of studied groups. Note the growth curves of F_1_ (N = 19) and F_2_ (N = 11) hybrids which are between the parental species’ curves.

The growth parameters significantly differed among the parental species of M and D and their F_1_ hybrids (ANOVA: *a*: F_(3,116)_ = 31.980, P < 0.0001; *K*: F_(3,116)_ = 9.8493, P = 0.000008; *T*: F_(3,116)_ = 5.4302, P = 0.001571). The parental species differed in all growth parameters (all p < 0.01). This difference is in accordance with our prediction of the parental species’ genetic distinction. F_1_ hybrids differed in parameter *a* (p = 0.019977 and 0.005097 in comparison with M and D, respectively). Moreover, F_1_ hybrids also differed in parameter *K* in comparison with D (p = 0.006029). The inflexion point *T* was similar in F_1_ and F_2_ hybrids in comparison with the parental species. The course of body growth of the parental species and F_1_ and F_2_ hybrids is shown in Fig. 5.

Hybridization of W with D also revealed distinctiveness of the parental species. Growth parameters of F_1_ and F_2_ hybrids were intermediate compared with values among the parental species (see Table 1).

## Discussion

Leopard geckos of the genus *Eublepharis* are long-living animals (maximum lifespan > 25 years, personal observation) laying multiple clutches per season. The clutches are of an invariant size, each consisting of two eggs, which are extraordinarily large compared to the maternal body^35,55^. This place their life-history strategy close to the “slow” end of the “fast-slow” continuum reported in lizards. Our analyses uncovered strong differences in growth trajectories among the examined species which were clearly associated with the asymptotic body weight. This suggests that the examined species/populations still significantly differ in their position along the fast-slow axis.

A three-parameter logistic regression model fits our long-term data covering the course of ontogeny from hatching to adulthood very well. Parental species/populations (A, M, W and D) differed significantly in the estimated asymptotic body weights and growth rates (except the growth rate and inflexion point, which were similar for two closely related populations of the yellow and white form of *E. macularius*).

We found that the growth parameters estimated by the logistic regression model were inter-correlated. Asymptotic body weight is tightly predicted by parameter *T*. Given the mutual relationship of growth parameters, we decided to compute separately the growth rate expressed as absolute and relative body weight increments. This approach allowed us to compare real increments of studied species/populations across the ontogeny (Fig. 3) and revealed similar results as those deduced from the estimates of parameter *K*. The growth rate was counterintuitively the lowest in large *E. angramainyu* (A) and highest in small *E. sp*. (D). The final body size was determined by the inflexion point parameter (*T*). The inflexion point reflects that the duration of the exponential growth period increases with mean asymptotic body size and thus easily overcompensates the effect of decreasing growth rates in the larger-bodied species.

A general life-history relationship described as the Rosa Lee phenomenon^56,57^ may provide an explanation for the decrease in the growth rate parameter (*K*) with asymptotic body size (*a*) found in our data set. Lee’s studies concerning age and growth determination in fishes demonstrated that individuals in a population with slower growth rates suffer less from mortality when they are young, which points to the existence of a trade-off between the growth rate and survival. This phenomenon was traditionally examined in fishes^58–61^ and only exceptionally applied to other vertebrates^62–65^. If further proved in geckos, large-bodied species might be selected to avoid mortality risk by reducing the growth rates. A preliminary inspection of our unpublished data sets suggests that within each species/population, the fast-growing individuals of the leopard gecko tend to suffer equal or even lower rates of juvenile mortality. However, the mortality pattern under laboratory conditions substantially differs from that under natural conditions. Thus, we have to search for alternative explanations for the reported reduced growth rates in larger geckos. Metabolic rates may be slightly constrained, e.g., by a positive allometric relationship between erythrocyte size and body size, which has been demonstrated in lizards including the eublepharid geckos^38,66^. Body growth of the whole organism, as well as the growth of particular organs and body segments, is orchestrated by hormones, growth factors and cell-cycle regulations^67^. These represent fundamental proximate mechanisms controlling growth rate and final body size. Beside growth hormones, there are sexual steroids, which are reported either to stimulate or to inhibit body growth. This was demonstrated especially in sexually dimorphic species^68–73^. Recent studies revealed that the deceleration of body growth is caused by the suppression of cell proliferation and is driven primarily by local rather than systemic mechanisms^67^.

Clear differences detected among the examined species/populations can be interpreted in terms of the “fast – slow” continuum of life-history strategies. *E. angramainyu* is a large-bodied species with a slow growth rate. This species attained the body weight close to asymptotic values at the age of about three years, but the first copulation was recorded at the age of five years. On the contrary, *E. macularius* is smaller, grows more slowly, and matures earlier (1–2 years). The sexual maturation is not known from the nature, but it is reasonable to suppose that it takes more time due to seasonality. However, the clutch size is invariant in eublepharid geckos and the relationship between body size and egg size is isometric^35^.

A similar analysis of the growth parameters is ideal for the comparison of parental and descendant individuals in the experimental crossing of the species/populations. It may also contribute to our knowledge of the influence the hybridization has on fitness, viability and competitiveness of F_1_ and F_2_ hybrids and backcrosses. Both positive and negative effects of hybridization are discussed in current literature^52,74–77^. A heterosis effect accompanying hybridization is traditionally used in agriculture and aquaculture because of an increased vigour (e.g., larger body size, faster growth rate, higher reproductive output, enhanced tolerance to environmental conditions). Similar experiments dealing with the effect of hybridization on body growth in squamate reptiles are completely missing. A pioneering study of hybridization among species of the genus *Lacerta*^54^ provided the first insight into the issue concerning hybridization in reptiles. Rykena reported intriguing data concerning hatchability, survival, fertility, and physical deformities of F_1_ and F_2_ hybrids and backcrosses^54^. The lacertids have genetic sex determination (GSD)^78^ and conclusions concerning the hybridization of GSD species may differ from those with temperature sex determination (TSD). Species with TSD are more abundant and are considered to be ancestral within the squamate reptiles^79^. However, information about the patterns of hybridization in TSD species is completely missing^47^.

Our study is a continuation of a long-term project dedicated to experimental hybridization of eyelid geckos. *E. macularius* belongs among the species with TSD^80^. Although sex determination of the *E. angramainyu* has never been experimentally tested, we expect it to be TSD as well, similarly like the closely related *Hemitheconyx caudicinctus*^79^. Moreover, the analysis of the karyotype in eyelid geckos revealed the absence of sex chromosomes^81^, which implies that the genome is the same in both sexes.

Preliminary results concerning the fitness indicators of F_1_ and F_2_ hybrids and backcrosses were published by Jančúchová-Lásková and her colleagues^43^. It was demonstrated that M is able to hybridize with congeneric A and produces viable and fertile hybrids without apparent malformations. Moreover, the introgression of the *E. angramainyu* genes into the *E. macularius* genome is possible via backcrossing. To examine the real competitiveness of hybrids and backcrosses with parental species and consequent advantages of hybridization, the observation of growth parameters is crucial. Hybridization produces novel genotypes that may be able to outperform their parental species and persist in unoccupied niches if necessary. The individual fitness and the extent to which hybrids interact with their parents (e.g., assortative mating or differential habitat use) is essential for evolutionary consequences of hybridization.

Our results of growth parameters revealed that F_1_ hybrids are an intermediate form between the parental species (Fig. 4). The body growth of only one F_2_ hybrid was very poor with asymptotic body size smaller than the parental yellow population of *E. macularius* (M). Poor fitness of this F_2_ hybrid is in congruence with the whole poor hatchability (6%) and viability (25%) of F_2_ hybrids demonstrated previously^43^. The growth parameters unequivocally corroborated that the putative fitness losses affect more hybrids of F_2_ generation, which is in accordance with the general Dobzhansky-Muller incompatibilities^82–85^ and empirical evidence^86^. MAxM and MxMA backcrosses had better hatchability and survival rate than F_2_ hybrids. The egg hatchability also dramatically differed between MAxA backcross and MAxM and MxMA backcrosses. While the latter one was possible to incubate (for details, see^43^), the hatchability of the opposite backcrosses (MAxA) was zero, even though the females laid eggs regularly. This fact again points to some genetic incompatibilities.

Concerning the growth parameters, backcross MAxM did not differ in the asymptotic body size and growth rate from the MxMA backcross. The only difference was in the timing of the deceleration of the body growth. These backcrosses attained larger body size than the parental yellow population of *E. macularius*. In this case, the effect of hybridization on body growth was positive. It is expected that the advantages of hybridization for backcrosses exert in an increased heterozygosity, avoidance of the inbreeding depression and more successful occupation of new habitats^87^.

Parental species of the yellow population of *E. macularius* and dark *E. sp*. differed in all of the growth parameters (all p < 0.01). Such a difference is in accordance with our prediction of genetic distinctiveness of these parental species. Hybridization of these two species reveals a similar effect on F_1_ hybrids. The growth parameters *a* and *T* were intermediate, but the growth rate was close to the smaller *E. sp*. in F_1_ hybrids. The success rate of gaining F_2_ hybrids was better than in the hybridization of M with A. The growth parameters of F_2_ hybrids were close to the F_1_ hybrids. The distribution of *E*. *macularius* and *E. sp*. is most probably allopatric, but the sequence divergence is not as striking as in M vs. A. The growth parameters of backcrosses (MxMD) were not monitored; nevertheless, the egg hatchability was high (92%, Landová *et al*., in prep.)

The crossing of the white population of *E. macularius* (W) with dark *E. sp*. revealed intermediate values of growth parameters in F_1_ and F_2_ hybrids in comparison with the parental species. It was not possible to test exactly the differences among the F_1_ and F_2_ hybrids due to the low number of hybrids, but the mean values of estimated growth parameters were comparable. Substantial differences in the results of hybridization of M/W with D were probably caused by the body size of the parental species (i.e., the body size of W is very similar to D).

In conclusion, we demonstrated that the growth parameters revealed by the three-parameter logistic regression model described the pattern of body growth of the studied species/populations of leopard geckos well. The pattern of body growth supports the “fast-slow” life-history continuum with species growing slowly but attaining large asymptotic body size and vice versa. Based on estimated growth parameters, it is possible to distinguish among these species/populations. We used this approach to study the effect of hybridization on fitness. We enriched our knowledge concerning the ability to hybridize in distinct species of the genus *Eublepharis*, which was previously observed in long-term geographic and evolutionary separated species of *E. macularius* and *E. angramainyu* by the additional experimental crossing of *E. macularius* with *E. sp*. The current approach tested competitiveness of F_1_ and F_2_ hybrids and backcrosses by comparison of their body growth parameters. Our results revealed that growth parameters are intermediate in both F_1_ hybrids. The poor fitness of F_2_ hybrid (MAxMA) is corroborating the outbreeding depression usually observed in F_2_ and other segregating generations of between-species hybrids. However, the introgression of A genes into M genome is enabled via backcrossing. This fact employs natural hybridization into the concept of species adaptation and speciation. A similar pattern concerning the occurrence of fertile hybrids of distinct species may be also expected in other taxa of Squamata.

## Methods

### Experimental animals and their maintenance

In the breeding stock of parental species, there were 51 *E. macularius* individuals of the yellow population (M), 40 *E. macularius* individuals of the white population (W), 6 individuals of large-bodied *E. angramainyu* (A), and 39 individuals of *E. sp*., which we further refer to as a dark population (D). Authors of the previous studies examining D geckos of the same stock considered the description of *E. fuscus* (Börner, 1981), and referred to this taxon, which is closely related to *E. macularius* sensu stricto, as *E. cf. fuscus*^38,39,88^.

The distribution of *E*. *macularius* covers large territories of Afghanistan, Pakistan and India^89^, althought a detailed distribution of the white and yellow form is not available. The B, W, and D populations were imported directly from unknown localities in Pakistan. *E. angramainyu* is native to Mesopotamia and SW Iran^90^. M and A are allopatric, their territories are separated by the Iranian Plateau and Zagros Mountains that are at least several million years old^89^. Thus, there was a long-lasting geographical isolation between *E. macularius* complex and *E. angramainyu* (cf. great sequence divergences between mitochondrial genes; uncorrected p-distances for 303 bp fragment of cyt b gene exceed 19%; Palupčíková, unpublished data).

All experimental parental species (M, W, D, and A) were the first generation of descendants of wild-caught animals. Parental A individuals were wild-caught as well, a putative locality of origin is Choqa Zanbil, Khuzestan province, Iran, 32“00’N 48'31’E, for more details about the locality see^91^.

The adult animals were housed individually in glass terrariums (60 × 30 × 20 cm or 30 × 30 × 20 cm, according to their body size). The floor of each cage was covered with bark substrate. Feeding and drinking dishes, as well as paper shelters, were provided. During the laying season, containers with adequately humid coconut substrate for egg deposition were added. The geckos were fed crickets and mealworms dusted with vitamins and minerals (Nutri Mix) weekly; AD_3_ and E vitamins were provided once per 14 days. The ambient temperature in the breeding room was about 28 °C with the permanent presence of basking sites in every terrarium to maintain a temperature gradient. During the egg-laying season (February to September), we checked the egg-deposition containers three times per week. The eggs were placed in the incubator and temperature was set to 28 ± 0.5 °C, which is the optimal and preferred temperature of *E. macularius*^32,92,93^. The incubation temperature of *E. angramainyu* was set lower (26 ± 0.5 °C) according to our previous experience with this species’ incubation. The hatchlings were housed individually in plastic boxes (20 × 20 × 15 cm) and were fed exclusively with crickets dusted with vitamins up to the three months of age.

To acquire F_1_ hybrids, females of the selected parental species/population were allowed to copulate with one breeding male of the second parental species/population. The resulting F_1_ hybrids were reared to sexual maturity and further bred to obtain F_2_ hybrids and/or backcrosses with either of the parental species. As the geckos of the studied genus *Eublepharis* can store sperm for several months, each experimental female was allowed to copulate exclusively with a single male during a given mating season (lasting from January/February to July/August). In contrast, males could copulate with multiple females within a single breeding season. From this hybridization, we got 68 viable F_1_ hybrids, 36 F_2_ hybrids, and 27 backcrosses.

All the individuals were weighed regularly by a digital balance to the nearest 0.01 g initially once per week (up to the age of 5 months), subsequently twice a month and later only once a month.

We studied the following thirteen categories of parental species/populations, their hybrids, and backcrosses that are further referred to as follows (the abbreviations are given in parentheses; there is always an abbreviation for a female followed by the one for a male with a cross (x) between:

1. M - a parental generation of the yellow population of *E. macularius*, both parents belong to the yellow population of *E. macularius* (M);
2. W - a parental generation of the white population of *E. macularius*, both parents belong to the white population of *E. macularius* (W);
3. A - a parental generation of *E. angramainyu*, both parents belong to *E. angramainyu* (A);
4. D – a parental generation of the dark population of the genus *Eublepharis*, both parents belong to this dark population (D);
5. MxA - the first-generation hybrid (F_1_), the mother of the yellow population of *E. macularius* (M) and the father of *E. angramainyu* (A);
6. MxD – the first-generation hybrid (F_1_), a mother of the yellow population of *E. macularius* (M) and a father of the dark population of *E. sp*. (D) grouped together with the mother of the dark population of *E. sp*. (D) and the father of the yellow population of *E. macularius* (M), we did not examine the mother’s identity effect;
7. WxD – the first-generation hybrid (F_1_), a mother of the white population of *E. macularius* (W) and a father of the dark population of *E. sp*. (D);
8. MxW – the first-generation hybrid (F_1_), a mother of the yellow population of *E. macularius* (M) and a father of the white population of *E. macularius* (W) grouped together with a mother of the white population of *E. macularius* (W) and a father of the yellow population of *E. macularius* (M), we did not examine the mother’s identity effect;
9. MAxMA - the second-generation hybrid (F_2_), both parents are F_1_ hybrids of the yellow population of *E. macularius* and *E. angramainyu* (MxA);
10. MDxMD – the second-generation hybrid (F_2_), both parents are F_1_ hybrids of the yellow population of *E. macularius* and the dark population of *E. sp*. (MxD);
11. WDxWD – the second-generation hybrid (F_2_), both parents are F_1_ hybrids of the white population of *E. macularius* and the dark population of *E. sp*. (WxD);
12. MAxM – the first-generation backcross with the yellow population of *E. macularius*, a mother is an F_1_ hybrid (MA) and a father belongs to the yellow population of *E. macularius* (M);
13. MxMA – the first-generation backcross with the yellow population of *E. macularius* (reciprocal to 12), a mother belongs to the yellow population of *E. macularius* (M) and a father is an F_1_ hybrid (MA)

Experiments were performed in accordance with the Czech law implementing all corresponding European Union regulations and were allowed by institutional Animal Care and Use Committee of the Charles University in Prague, and approved by Ethical Committee of Ministry of Education, Youth and Sports of the Czech Republic license no. 18147/203 and 24773/2008–10001.

### Statistical methods

We applied a three-parameter logistic regression model (Equation 1) to analyse the growth trajectories of the overall sample of a given species, hybrids, backcrosses. We have previously found that this model^22^ fits very well the data covering the body growth of reptiles from hatching to adulthood^9^. We used the Levenberg-Marquardt algorithm (with 1000 maximum number of iterations), which minimized the sum of squares between the predicted and observed growth values.

1 $${\rm{Body}}\,{\rm{weight}}\,[{\rm{grams}}]={\rm{a}}/(1+{{\rm{e}}}^{-{\rm{K}}({\rm{age}}-{\rm{T}})})$$

Growth parameter *a* predicts the asymptotic body size, parameter *K* estimates the growth rate, and the last one, parameter *T*, expresses the age at the inflexion point (i.e., a place of the maximal growth rate where the growth curve changes from convex to concave and the individual growth rate starts decreasing). We set these parameters’ starting values as follows: *a* = 30, *K* = 0.005 and *T* = 150. Growth equations were computed separately for each individual. As the number of females (290) highly exceeded the number of males (35) and the intersexual differences were much smaller than the interspecific ones, we pooled the data of both sexes in distinct groups for a comparison between species, hybrids, and backcrosses.

Interspecific differences in growth curve parameters were tested by a general linear model (ANOVA, post-hoc Tukey’s test for unequal N). The effect of growth rate and inflexion point on asymptotic body weight was tested by multiple regression separately for each group. All calculations were performed using STATISTICA, version 6.0.

In addition to the comparison of growth parameters revealed by the logistic regression model, we compared body weight increments from real measurements calculated as an absolute body weight increment (aBWI = actual body weight - previous body weight) and a relative body weight increment (rBWI = aBWI/actual body weight).

### Data availability

All data generated or analysed during this study are included in this published article (and its Supplementary Information files).

## Electronic supplementary material

Dataset 1
